# Multi-region exome sequencing reveals the intratumoral heterogeneity of surgically resected small cell lung cancer

**DOI:** 10.1038/s41467-021-25787-x

**Published:** 2021-09-14

**Authors:** Huaqiang Zhou, Yi Hu, Rongzhen Luo, Yuanyuan Zhao, Hui Pan, Liyan Ji, Ting Zhou, Lanjun Zhang, Hao Long, Jianhua Fu, Zhesheng Wen, Siyu Wang, Xin Wang, Peng Lin, Haoxian Yang, Junye Wang, Mengmeng Song, Xin Yi, Ling Yang, Xuefang Xia, Yanfang Guan, Wenfeng Fang, Yunpeng Yang, Shaodong Hong, Yan Huang, Pansong Li, Yaxiong Zhang, Ningning Zhou

**Affiliations:** 1 Department of Medical Oncology, Sun Yat-Sen University Cancer Center, State Key Laboratory of Oncology in South China, Collaborative Innovation Center for Cancer Medicine, Guangzhou, China; 2 Department of Thoracic Surgery, Sun Yat-Sen University Cancer Center, State Key Laboratory of Oncology in South China, Collaborative Innovation Center for Cancer Medicine, Guangdong Esophageal Cancer Institute (GECI), Guangzhou, China; 3 Department of Pathology, Sun Yat-Sen University Cancer Center, State Key Laboratory of Oncology in South China, Collaborative Innovation Center for Cancer Medicine, Guangzhou, China; 4 Department of Clinical Research, Sun Yat-Sen University Cancer Center, State Key Laboratory of Oncology in South China, Collaborative Innovation Center for Cancer Medicine, Guangzhou, China; 5 Geneplus-Beijing Institute, Beijing, China; 6 Department of Thoracic Surgery, Sun Yat-Sen University Cancer Center, State Key Laboratory of Oncology in South China, Collaborative Innovation Center for Cancer Medicine, Guangzhou, China

**Keywords:** Cancer genomics, Small-cell lung cancer

## Abstract

Small cell lung cancer (SCLC) is a highly malignant tumor which is eventually refractory to any treatment. Intratumoral heterogeneity (ITH) may contribute to treatment failure. However, the extent of ITH in SCLC is still largely unknown. Here, we subject 120 tumor samples from 40 stage I-III SCLC patients to multi-regional whole-exome sequencing. The most common mutant genes are TP53 (88%) and RB1 (72%). We observe a medium level of mutational heterogeneity (0.30, range 0.0~0.98) and tumor mutational burden (TMB, 10.2 mutations/Mb, range 1.1~51.7). Our SCLC samples also exhibit somatic copy number variation (CNV) across all patients, with an average CNV ITH of 0.49 (range 0.02~0.99). In terms of mutation distribution, ITH, TMB, mutation clusters, and gene signatures, patients with combined SCLC behave roughly the same way as patients with pure SCLC. This condition also exists in smoking patients and patients with EGFR mutations. A higher TMB per cluster is associated with better disease-free survival while single-nucleotide variant ITH is linked to worse overall survival, and therefore these features may be used as prognostic biomarkers for SCLC. Together, these findings demonstrate the intratumoral genetic heterogeneity of surgically resected SCLC and provide insights into resistance to treatment.

## Abstract (web summary)

Multi-region sequencing of small cell lung cancers (SCLC) can improve our understanding of the disease. Here the authors analyse 120 multi-region samples from 40 SCLC patients with whole exome sequencing and characterise their mutational burden, evolution, heterogeneity, and potential prognostic biomarkers.

## Introduction

Lung cancer is the most prevalent cancer in the world, with 15% of patients diagnosed with the highly aggressive and metastatic malignancy small cell lung cancer (SCLC)^1^. About one-third of SCLC patients present with limited disease (LD) and the remaining patients are diagnosed with extensive disease (ED) SCLC at the time of initial diagnosis. The 5-year overall survival (OS) rate for ED SCLC is below 7%^2^. For SCLC patients, there has been no significant progress in the treatment modalities over the past decade. While the vast majority of patients are sensitive to chemotherapy and radiotherapy at the time of the initial treatment, all patients inevitably face the dilemma of chemoresistance and disease progression^3^. Recently, immunotherapy was approved for the comprehensive treatment of ED SCLC^4–8^. Yet, recurrence, drug resistance, and cancer as the cause of death are still common in the course of SCLC. How to improve a patient’s prognosis remains an unmet need for this recalcitrant malignancy.

An important factor in the failure of anticancer treatment is intratumor heterogeneity (ITH), which refers to distinct tumor cell populations (with different molecular and phenotypic profiles) within the same tumor specimen, resulting in differences in the tumor growth rate, invasion ability, drug sensitivity, and prognosis^9^. Next-generation sequencing (NGS) technology has been widely used for tumor genome variation research and has shown excellent capabilities in ITH research. For example, in the TRACERx (TRAcking Cancer Evolution through therapy (Rx)) lung study, multi-region sampling of lung cancer tissues from 100 early stage non-small cell lung cancer (NSCLC) patients using multi-region whole-exome sequencing (MRS) revealed ubiquitous ITH in patients and copy number variation (CNV). ITH was associated with prognosis, which provides a reference for subsequent cancer genome research^10^. Elucidating the heterogeneity of SCLC could help better our understanding of disease management. A recent study found that chemotherapy caused increased ITH, leading to the development of multiple mechanisms of drug resistance in ED SCLC^11^. However, the ITH of LD SCLC patients without chemotherapy remains unknown due to a lack of tumor samples.

In this study, we aim to provide the intratumoral genetic heterogeneity landscape of surgically resected SCLC, by analyzing the whole-exome sequencing data of 120 samples from 40 patients with SCLC. We characterize their mutational burden, heterogeneity, evolution, and potential biomarkers. Considerable intratumoral genetic heterogeneity is present among SCLC. We further identify several heterogeneity-related prognostic biomarkers.

## Results

### Patients’ characteristics

We included 40 surgically resected SCLC patients in this study, among them, 6 were diagnosed with combined SCLC (C-SCLC). Most SCLCs (34/40) were pure SCLC (P-SCLC). Table 1 shows the patients’ clinical characteristics. The median age was 62 years old. Most patients were male (35, 87.5%) and had a history of smoking (31, 77.5%). All patients underwent surgery, with a median tumor size of 22.5 mm. About 65% of patients received further treatment after surgery. Fifteen patients (15, 38%) died after a median follow-up time of 22.82 months.

**Table 1:** Clinical characterization of our SCLC cohort.

Characteristics	Total (*n* = 40)	P-SCLC (*n* = 34)	C-SCLC (*n* = 6)
Median age in years (range)	62 (23–76)	64 (23–75)	63 (50–76)
*Sex (%)*			
Male	35 (87.5%)	31 (91%)	4 (66%)
Female	5 (12.5%)	3 (9%)	2 (33%)
*Smoking (%)*			
Non-smoker	8 (20%)	5 (15%)	3 (50%)
Smoker	31(77.5%)	28 (82%)	3 (50%)
NA	1 (2.5%)	1 (3%)	0 (0%)
*Drinking (%)*			
Never	17 (42.5%)	13 (38%)	4 (66%)
Drinking	22 (55%)	20 (59%)	2 (33%)
NA	1 (2.5%)	1 (3%)	0 (0%)
Median tumor size (range)	22.5 (1.6–420)	22.5 (1.6–420)	22.83 (2.4–54)
*Stage (%)*			
I	15 (37.5%)	12 (35%)	3 (50%)
II	7 (17.5%)	5 (15%)	2 (33%)
III	18 (45%)	17 (50%)	1 (16%)
IV	0 (0%)	0 (0%)	0 (0%)
*Treatment after surgery (%)*			
Yes	26 (65%)	23 (68%)	3 (50%)
No	14 (35%)	11 (32%)	3 (50%)
*Status (%)*			
Alive	25 (62%)	20 (59%)	5 (83%)
Dead	15 (38%)	14 (41%)	1 (16%)

### Mutation landscape of 40 SCLC patients using multiple-regional sequencing

**Fig. 1:**
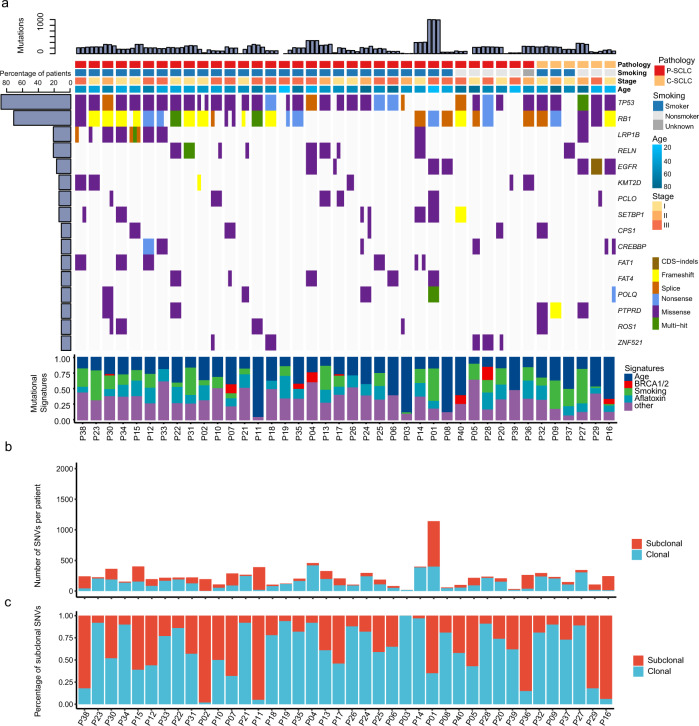
Mutational spectrum of SCLC. **a** Mutational landscape of SCLC (*n* = 40). Mutated gene frequency >15% involved in previously reported significant mutated genes in SCLC are shown for each region of the individual patient. Upper, TMB count; middle, heatmap for driver mutations; lower, mutational signatures. **b** Counts in clonal and subclonal mutations for each patient (*n* = 40). **c** Percentage of subclonal mutations for each patient (*n* = 40). SCLC small cell lung cancer, P-SCLC pure small cell lung cancer, C-SCLC combined small cell lung cancer, SNVs single-nucleotide variants, CDS coding sequence.

We subjected 120 formalin-fixed paraffin-embedded (FFPE) SCLC samples (3 regions per patient) to MRS. In total, 33,153 non-silent somatic mutations were identified with an average 252× sequencing depth (Supplementary Data 1). We found an average of 340 mutations (range 33–1552) from multi-region for each patient. The median multi-region based tumor mutation burden (TMB) of SCLC was similar with single-region based TMB in our cohort and The Cancer Genome Atlas (TCGA) cohort (Supplementary Fig. 1a, Mann–Whitney–Wilcoxon test, both *p* > 0.05). There was a positive correlation between TMB and tumor neoantigen burden (TNB) (Spearman’s correlation coefficient, *r* = 0.59, *p* < 0.001; Supplementary Fig. 1b). The most frequent mutant genes were *TP53* (88%) and *RB1* (72%), which were clonal mutations; while *LRP1B* (22%), *PCLO* (15%), and *KMT2D* (15%) were subclonal mutations (Fig. 1a, Supplementary Fig. 2c, Supplementary Data 2). The C > T and C > A transversions were enriched in these patients (Supplementary Fig. 1c, d). The age-associated, *BRCA1/2*-associated, tobacco-associated, and aflatoxin-associated signatures were also major mutational signatures in these patients (Fig. 1a). The age-associated, aflatoxin-associated, and DNA repair-associated signatures were the top signatures in the branch, while the age-associated and smoking-associated signatures were major ones in the trunk (Supplementary Fig. 1e, f).

Non-silent mutation distribution showed ITH in patients with SCLC varied significantly (Fig. 1b). Percentages ranged from 17 to 100% (Fig. 1c). We found a medium mutational heterogeneity (0.30, quartile 0.12–0.56) in our SCLC cohort, and the SNV ITH of P-SCLC and C-SCLC were not significantly different with NSCLC of TRACERx study (*p* = 0.065 and *p* = 0.32)^10^ (Fig. 1c and Supplementary Fig. 2b). We also showed the distribution of mutations in ten common oncogenic signaling pathways^12^ (Supplementary Fig. 2g) and identified that mutations in the *TP53* and *RTK-Ras-ERK* signaling pathways were predominantly clonal mutations.

### Intratumoral heterogeneity in CNV

**Fig. 2:**
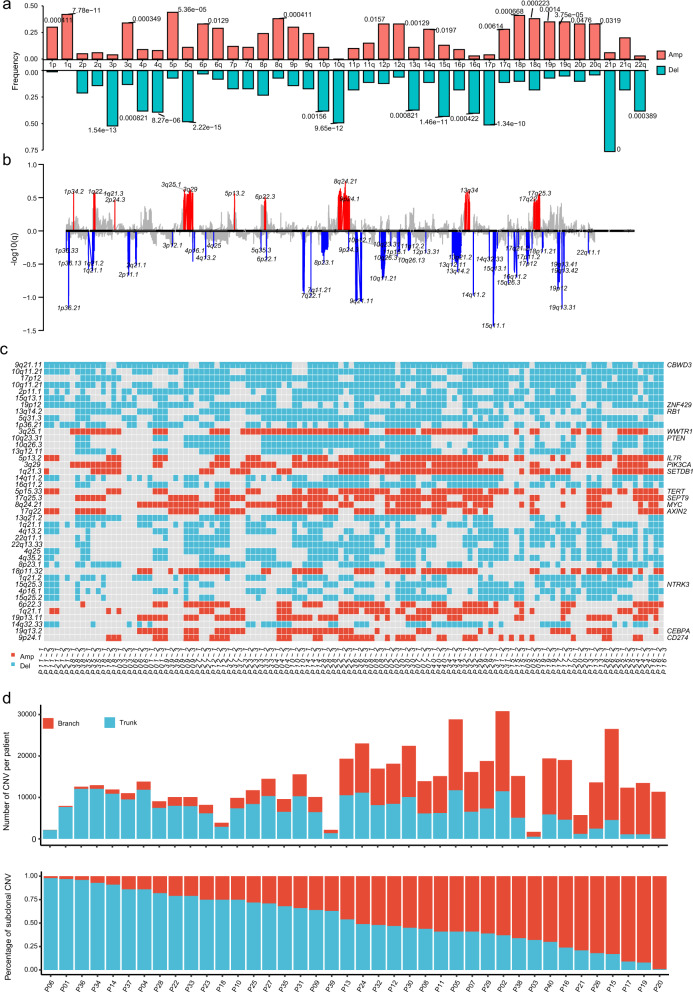
Copy number alterations in our cohort. **a** Arm level CNVs identified by GISTIC2.0 in SCLC (*n* = 40). False discovery rate (FDR) corrected *p* value represents significant changes from Benjamini–Hochberg testing. **b** The genome chromosome plots depict significant cytobands identified by GISTIC2.0. **c** The significant somatic focal CNVs of pure SCLC and combined lung cancer are shown for each region of the individual patient. Cytobands with genes involved in cosmic drivers and those that occurred in at least 50% of patients are shown. **d** Counts in the trunk and branch of CNVs for each patient; Percentage of branch CNVs for each patient (*n* = 40). SCLC small cell lung cancer, Amp amplification, Del deletion, CNVs copy number variations.

SCLC exhibited somatic arm-level CNV alterations including amplification at chromosomes 1, 12, 18, 19, 20, 3q, 5p, 6p, and 8q, and deletions at chromosomes 4, 10, 3p, 5q, 13q, 15q, 16q, 17q, 21p, and 11q (Fig. 2a, Supplementary Data 3–5). Significantly amplified regions included 1p34.2 (*HEYL*), 1q21.3 (*APH1A*), 2p24.3 (*MYCN*), 3q29 (*PIK3CA*), 5p13.2 (*IL7R*), 6p22.3 (*E2F3*), 8q24.21 (*MYC*), and 9p24.1 (*CD274*, *PDCD1LG2*) as well as deleted regions 3p12.1, 4q13.2, 5q35.3, 9q21.11(*CBWD3*), 10q23.31 (*PTEN*), 13q14.2 (*RB1*), 14q11.2, 15q25.3 (*NTRK3*), 19p12 (*ZNF429*), and 22q11.1 (Fig. 2b, c). Using CNV ITH, a median of 0.485 (range 0.02–0.99 per sector) was found in SCLC (Fig. 2d). Among them, *IL7R*, *PIK3CA*, *SETDB1*, *TERT*, *SEPT9*, *MYC*, *CEBPA*, and *CD274* genes were amplified as frequently recurring clonal genes, while the clonal depleted genes like *CBWD3*, *RB1*, and *PTEN* were identified in our patients (Supplementary Fig. 2e).

### Clonal evolution and pathway enrichment

**Fig. 3:**
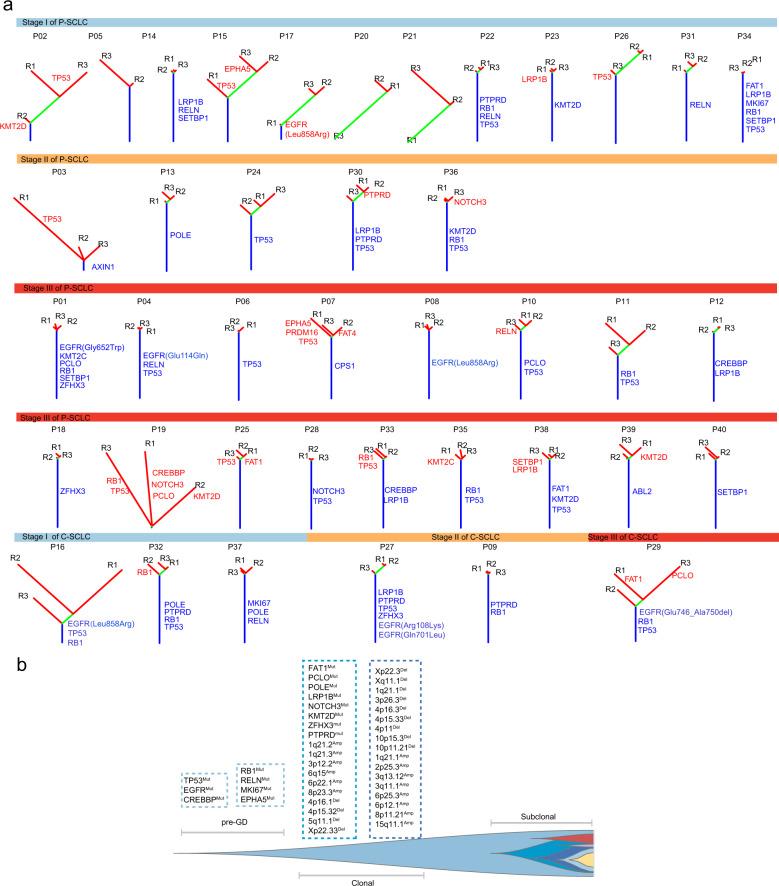
Phylogenetic trees and evolution in SCLC. **a** Phylogenetic trees for each patient (*n* = 40) stratified according to stages. **b** The evolution mode in all patients (*n* = 40). P-SCLC pure small cell lung cancer, C-SCLC combined small cell lung cancer, pre-GD pre-genome doubling.

**Fig. 4:**
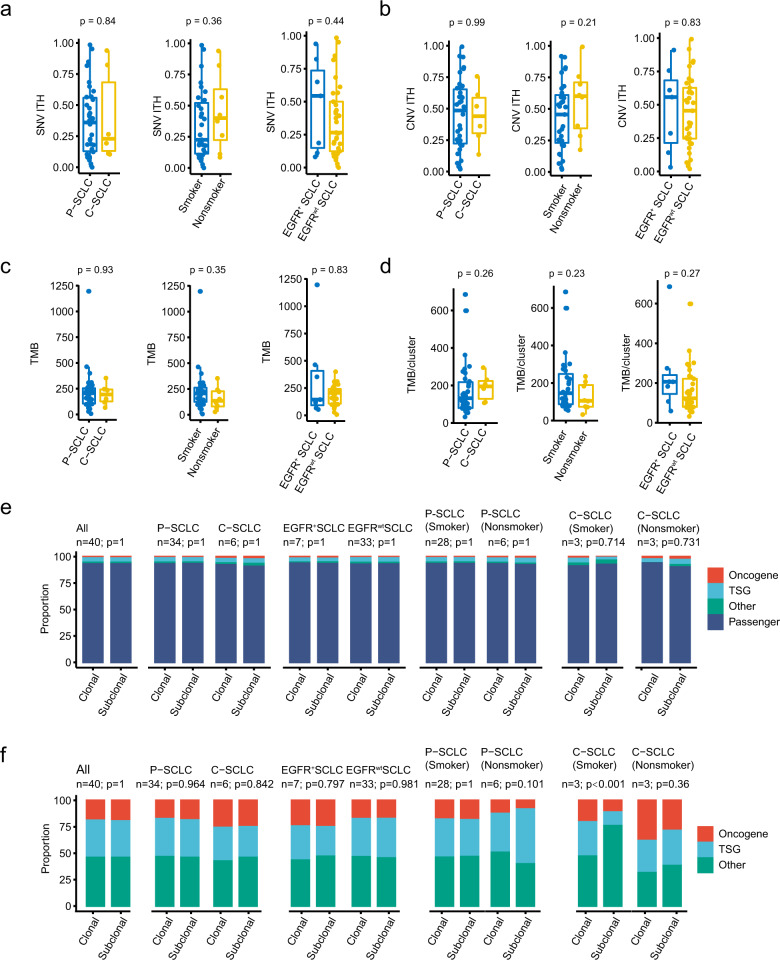
The ITH and clinicopathological characteristics of SCLC. The comparison of **a** SNV ITH, **b** CNV ITH, **c** TMB, **d** average TMB per cluster between pure SCLC (*n* = 34) and combined lung cancer (*n* = 6), EGFR mutant (*n* = 7), and wild type (*n* = 33), as well as smoking (*n* = 31) and nonsmoking (*n* = 8) subgroups. *p* Value from two-sided Mann–Whitney *U* test. Boxplots are represented by a centerline, median; box limits, the 25th and 75th percentiles; whiskers extend represent the lower and upper values within 1.5 * inter-quartile range. **e**, **f** The proportion of driver genes, passenger genes, and other genes in the trunk and branch. *p* Value from two-sided Fisher’s exact test. SNV single-nucleotide variant, CNV copy number variation, ITH intratumoral heterogeneity, P-SCLC pure small cell lung cancer, C-SCLC combined small cell lung cancer, TMB tumor mutation burden, TSG tumor suppressor gene.

We also constructed phylogenetic trees based on somatic mutations detected in multiple regions. Figure 3a shows the phylogenetic tree for each patient according to their disease stage. In particular, *TP53*, *EGFR*, and *CREBBP* mutations were common early clonal events involved in the evolution of SCLC (Fig. 3b), while *RB1* and other mutations were late clonal events. Generally, among clonal and subclonal mutations, passenger mutations were proportionally higher than driver mutations (oncogene and TSG, Fig. 4e).

### Correlation between genetic alterations and clinical characterization

No significant relationship was observed between ITH and other clinical variables, including pathology, smoking history, *EGFR* mutation status, and tumor stage (Fig. 4a, b, Supplementary Fig. 2d). Among the *EGFR* mutations, three patients carried non-classic *EGFR* mutations (p.G652W, p.E114Q, p.Q701L|p.R108K; Supplementary Data 6) and four had classic mutations (p.L858R and EX19del). Classic *EGFR* mutations were found in two (5.9%, 2/34) P-SCLC and two (33%, 2/6) C-SCLC patients, respectively. In our cohort, we found that all *EGFR* mutations co-occurred with *TP53* inactivation and *RB1* inactivation (mutation and/or loss) (Supplementary Data 6). The *TP53/RB1/EGFR* mutations were independent of clinical (tumor stage and tumor size), and genomic features (TMB, ITH, and WGD) in SCLC (Supplementary Fig. 6a). Intriguingly, *EGFR/RB1/TP53*-mutant patients exhibited higher ploidy than those with wild-type (*p* = 0.017). And WGD occurred in all of the *EGFR/RB1/TP53* mutant patients (Supplementary Fig. 6a). Besides, these mutations were not associated with disease-free survival (DFS) or OS in the absence or presence of treatment after surgery (Supplementary Fig. 6b, c).

**Fig. 5:**
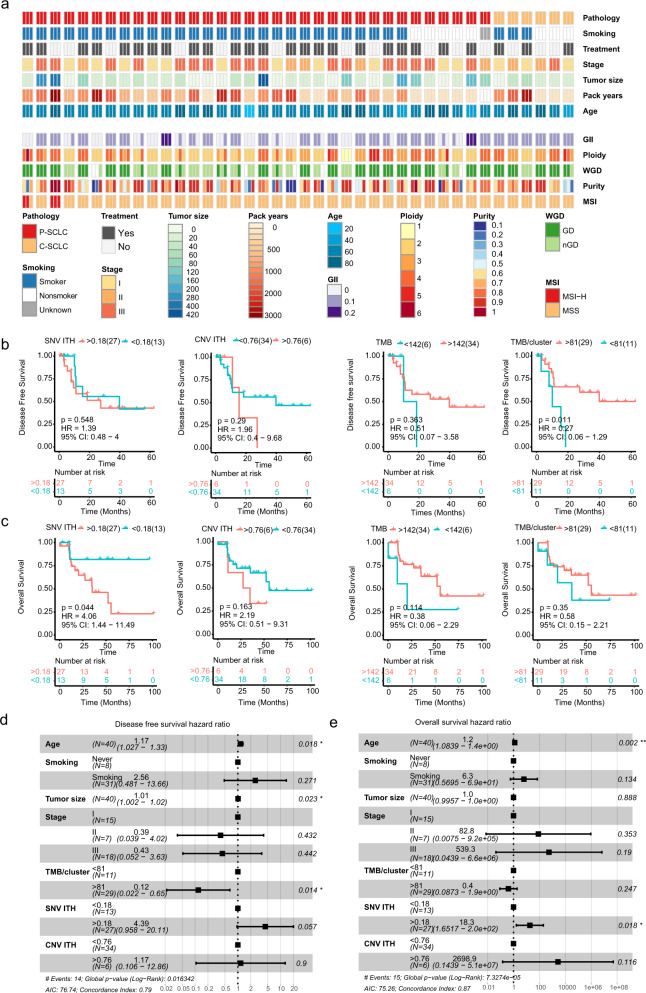
The relationship between heterogeneity and clinical characterization in SCLC. **a** A heatmap displaying the clinical information and genomic features for each patient (*n* = 40). The Kaplan–Meier plot depicts the estimation of disease-free survival (**b**) and overall survival (**c**) with parameters including SNV ITH, CNV ITH, mutation cluster, and TMB per cluster. The *p* value and hazard ratio were determined using the two-sided log-rank test. The forest plot showing multiple covariate Cox regression analysis of disease-free survival (**d**) and overall survival (**e**) by subgroups including age, smoking, tumor size, stage, and ITH in SCLC. A two-sided, unpaired, Wilcoxon rank test was performed for the statistical comparison among subgroups. WGD, whole-genome duplication; GII genome instability index, MSI microsatellite instability, SNV single-nucleotide variant, CNV copy number variation, ITH intratumoral heterogeneity, P-SCLC pure small cell lung cancer, C-SCLC combined small cell lung cancer, TMB tumor mutation burden, HR hazard ratio, CI confidence interval.

Supplementary Fig. 3 and Fig. 5a show the basic clinicopathological information in this cohort. Patients with P-SCLC/C-SCLC, smoker/non-smoker, *EGFR* mutant/wild type had similar levels of ITH, TMB, and mutation clusters, and they exhibited no discrepancy in their gene signature and mutation landscape (Fig. 4, Supplementary Fig. 4b, c). Remarkably, a higher TMB/cluster correlated with better DFS using univariate analysis, while the SNV ITH was correlated to OS (Fig. 5b, c). However, no significant correlation was observed among DFS or OS and TMB, mutation cluster, or tumor stage (Fig. 5b, c, Supplementary Fig. 6d, e). In a multivariate analysis adjusted for age, tumor size, tumor stage, and smoking status, only TMB/cluster were associated with better DFS, and SNV ITH is also linked to worse OS of SCLC (Fig. 5d, e).

All the cases with recurrence received systemic chemotherapy in our cohort. No ITH discrepancies were observed in patients according to the recurrence status and systemic chemotherapy (Supplementary Fig. 6f). ITH and TMB/cluster were not associated with survival outcomes in the recurrent cases (*p* > 0.05, *n* = 11, Supplementary Fig. 6g). Cases that received systemic chemotherapy had a superior overall outcome (Supplementary Fig. 6g), suggesting the favorable role of chemotherapy after surgery in the treatment of SCLC.

## Discussion

Many SCLC patients are sensitive to initial treatment, but all patients inevitably face the dilemma of chemoresistance. It has been speculated that ITH is common in treatment-naive SCLC, with many drug-resistant subclones^13^. Yet, because of the lack of available tumor samples, this gap is still vacant in the field of SCLC research. Moreover, research in the field has mainly utilized traditional genomic sequencing of a single site which is unable to capture the full genomic landscape^14^. Whereas MRS is superior in evaluating the ITH of SCLC. Therefore, we performed MRS in a cohort of surgery resected SCLC patients. There was widespread ITH in SNV and CNV in SCLC, with a medium ITH score among different patients. Such universal ITH indicates a complex genomic landscape of SCLC even at the early stage and illustrates the dilemma of current treatment, such as rapid disease progression and relapse with refractory disease.

For the somatic mutations, *TP53* and *RB1* had the highest mutation frequency^15^. This corresponds with current research. Previous single-region sequencing revealed extensive common cancer-specific genomic alterations in SCLC, such as *TP53* and *RB1*^16–18^. They are also the most common clonal mutations identified in the MRS data, namely, somatic genetic alterations of *TP53* and *CREBBP*, which were almost exclusively early clonal events. Most of the patients in our cohort carried subclonal mutations, including *LRP1B*, *KMT2D*, and *PCLO*, which appeared randomly in different regions. The same phenomenon occurred in the CNV events, however, not all CNV events existed in every tissue from the same sample. This highlights the limitations of single-region sequencing and emphasizes the advantages of MRS for better understanding the genomic landscape in precision medicine.

*EGFR* mutations are a rare occurrence in either de novo SCLC or in cases of transformed *EGFR*-mutant (*EGFR*-mt) adenocarcinoma^19^. In our study, the frequency of classic *EGFR* mutations in P-SCLC was 5.9%. These data were comparable with previous reports of 2.6% in Taiwanese and 2.0% in a Chinese cohort^19,20^. Our *EGFR*-mutant SCLC patients did not receive EGFR-TKI therapy, and *EGFR* mutation status is not associated with recurrence after surgery (Supplementary Fig. 3d). An *EGFR* mutation is considered an early clonal event in our analysis (Fig. 3b). However, a lower driver dominant *EGFR* score did not support its role as a driver gene in SCLC, which is distinct from common NSCLC (Supplementary Fig. 4a). In other words, an *EGFR* mutation was not a predominant driver gene in SCLC. Currently, there is no targeted therapy in *EGFR*-mutant SCLC. The majority of de novo *EGFR*-mt SCLC are resistant to EGFR-TKI therapy, compared with *EGFR*-mt NSCLC^21^, which may be due to focusing much more on the driver gene “*EGFR*” and neglecting of passenger mutations’ effect. *EGFR* passenger mutations may also collaborate synergistically with driver mutations to trigger tumorigenesis in SCLC. Previous researchers have shown that *EGFR/RB1*/*TP53* are key events that transform NSCLC to SCLC after EGFR-TKI treatment^22,23^. In our treatment-naive SCLC cohort, we also found that all *EGFR* mutations co-occurred with *TP53* and *RB1* mutations. *EGFR/RB1*/*TP53* mutant patients had WGD events and exhibited higher ploidy than those with wild-type (Supplementary Fig. 6a). Yet, the *TP53/RB1/EGFR* mutations were independent of clinicopathologic features and not associated with prognosis. Based on the tumor evolutionary algorithm model proposed by Swanton et al.^10^, we conferred that *TP53* and *EGFR* mutations were early events in the evolution of SCLC, while the *RB1* mutation and loss occurred later, indirectly suggesting a key role of *RB1* inactivation in SCLC evolution. However, this hypothesis needs validation in further studies.

We sought to explore the relationship between ITH scores and clinicopathological features. We were particularly interested in the six patients with C-SCLC in this study cohort. Comprehensive research showed that this group of patients behaved much in the same way as P-SCLC patients, both in terms of mutation distribution, ITH, TMB, mutation clusters, and gene signatures. This condition is also present in patients with *EGFR* mutations and those with a history of smoking. Among diagnosed SCLC patients, most patients have a history of smoking. We paid special attention to the evolutionary tree of non-smoking SCLC patients and found there was no obvious difference compared with smoker patients (Supplementary Fig. 5). To some extent, the intratumoral heterogeneity of the SCLC genome is independent of common clinicopathological features, such as pathological types, smoking history, and driver gene mutation status, but there is still a relatively uniform moderate level of intratumoral heterogeneity. A previous study reported widespread ITH in chemotherapy-treated SCLC and found that it may lead to poor treatment response and prognosis. We observed the same performance of SNV ITH in treatment-naïve LD SCLC patients. Multivariable COX analysis supported the independent prognostic role of SNV ITH for OS. We turned our perspective to another tumor heterogeneity assessment algorithm, TMB per cluster, which seems to be another potential prognosis biomarker. We found that more TMB per cluster is linked to early disease recurrence and progression. It indicated complex mutations inside the tumor may lead to the failure of anti-cancer treatment. Further research on its relationship with treatment sensitivity and resistance is needed.

Although our study presents several findings, there are several limitations. First, our results would have been more reliable with more patients from other centers. Related to our limited sample, we did not perform dynamic genome monitoring for each patient. We also did not provide a better understanding of the tumor microenvironment of SCLC. In addition, we should notice that the presence of technical noise in sequencing data is common, and genuine intratumor genetic heterogeneity is hard to distinguish from these sequencing artifacts^24^. It may lead to the overestimation of ITH. Therefore, we used two mutation calling algorithms and strict criteria to filtering out these private artifacts, and to minimize the impact of artifacts^25,26^. Due to the unavailability of the samples, we could not validate our results in the same sample. Nevertheless, further studies with high depth sequencing are required to accurately quantifying ITH.

We demonstrated the ITH landscape of surgically resected SCLC. Despite a moderate mutation burden, SCLC showed a medium intratumoral heterogeneity with high SNV and CNV ITH at the early stage, which may explain the difficult treatment dilemma faced by SCLC patients.

## Methods

### Patients and samples

Forty enrolled SCLC patients underwent thoracic surgery at Sun Yat-Sen University Cancer Center between September 2009 and September 2018. The diagnosis of SCLC was confirmed by two pathologists via immunohistochemistry. None of the patients received any previous systematic anti-cancer therapy. We collected 120 surgically resected FFPE tumor tissues from 40 patients (3 tumor regions in different quadrants for each patient). A paired peripheral blood sample was obtained during the surgery. The study protocol was approved by the institutional review board of Sun Yat-Sen University Cancer Center. We have complied with all relevant ethical regulations for work with human participants, and that written informed consent was obtained.

### Multi-region whole-exome sequencing

For each region of the patient, DNA was extracted from the FFPE kit (Promega) according to the manufacturer’s instructions. We constructed the sequencing libraries from native DNA using the xGen^®^ Exome Research Panel (Integrated DNA Technologies, Iowa, IA, USA) and the NEB Next Ultra DNA Library Prep Kit (Lot: NEB-0311611, NEB, UK) with a KAPA polymerase (KapaBiosystems, Wilmington, MA, USA). Whole-exome sequencing was performed using GeneSeq-2000 (Geneplus-Suzhou, Suzhou, China), with 100-bp paired-end sequencing. The data preprocessing and variant callings were based on the Sentieon-genomics pipeline (version sentieon-genomics-201808)^27^ with parameters as follows (*sentieon driver -t 16 -r hs37d5.fa -algo VarCal -v SNP.vcf -resource 1000* *G_phase1.snps.high_confidence.b37.vcf -resource_param 1000G,known* = *false,training* = *true,truth* = *false,prior* = *10.0 -resource 1000G_omni2.5.b37.vcf -resource_param omni,known* = *false,training* = *true,truth* = *false,prior* = *12.0 -resource hapmap_3.3_b37_pop_stratified_af.vcf -resource_param hapmap,known* = *false,training* = *true,truth* = *true,prior* *=* *15.0 -resource dbsnp_138.b37.del100.vcf.gz -resource_param dbsnp,known* *=* *true,training* *=* *false,truth* = *false,prior* *=* *2.0 -annotation QD -annotation MQ -annotation MQRankSum -annotation ReadPosRankSum -annotation FS -var_type SNP -plot_file SNP.varcal.plotfile -tranches_file SNP.varcal.tranches SNP.varcal.recal && sentieon driver -r hs37d5.fa -algo ApplyVarCal -v SNP.vcf -tranches_file SNP.varcal.tranches -var_type SNP -recal SNP.varcal.recal SNP.vqsr.vcf*). We removed the terminal adapter sequences and low-quality reads from the raw data with these filters (paired-end reads were removed if anyone read meet one of the three criteria: (a) half of bases with base quality ≤ 5; (b) the ratio of N bases exceeding 5%; (c) the average base quality below 0). The clean reads were aligned with the human reference genome (hg19) using BWA MEM (v0.7.17–r1188). LocusCollector and Dedup were used to mark and remove PCR duplicates. Realignment and recalibration were performed using a Sentieon-genomics Realigner. The peripheral blood monocyte cell DNA served as a control (germline).

### Somatic variant detection

Single nucleotide variants (SNVs) were called by Sentieon-genomics Tnscope (https://support.sentieon.com/appnotes/out_fields/#tnscope-reg) and MuTect2 software. Small insertions and deletions (indels) were identified by the Sentieon-genomics VarCall algorithm. High-quality reads were selected with a Phred score ≥30, a mapping quality score ≥30, and without paired-end reads bias. The candidate somatic mutations underwent the following filtering strategies: (i) the mutation was detected in at least five high-quality reads and supported by at least ten normal reads and the total depth was greater than 30 × at the loci in the tumor. (ii) the mutant allele had to be present in ≥3% of the variant allele frequency (VAF) identified by TNscope. (iii) the mutation was not present in >1% of the population in the 1000 Genomes Project (version phase 3), dbSNP databases (The Single Nucleotide Polymorphism Database, version dbSNP 138), and (iv) the local blacklist database. For somatic tumor mutations, if mutations were identified in one or two regions, we rescued these mutations in the rest region for each tumor. And the VAF of rescued mutations with greater than 1% was supported by fewer than five mutant reads in normal tissues. All these mutations were further filtered by the “PASS” output of MuTect2. The final overlapped variants were annotated using Ensembl Variant Effect Predictor (VEP v93.3) software^28^. The candidate variants were all manually verified in the Integrative Genomics Viewer (v2.3.66). Microsatellite instability (MSI) was calculated using a published MSIsensor tool (v0.2)^29^.

### Somatic CNV identification and tumor purity estimation

Somatic CNV was identified with FACETS (v0.5.11)^30^. Significant somatic CNVs were obtained using GISTIC2.0 with the output from FACETS^31^. CNVs gain was defined as segments with copy number/ploidy ≥ log2(2.5/2), while CNV loss was segmented with copy number/ploidy < log2(1.5/2). Whole-genome doubling was detected using modified McGranahan’s method^32^. Specifically, *p* values that were defined as the ratio of 10,000 simulated copy number events to the observed CNVs, then the whole genome doubling events were considered if *p* ≤ 0.001 for haploid or diploid or triploid; *p* ≤ 0.05 for tetraploid; *p* ≤ 0.5 pentaploid, and *p* ≤ 1 for multi-ploidy greater than six. The genome instability index (GII) was determined by the total length of gain plus the loss region divided by chromosome size^33^. Clonal gain demonstrated all regions of the tumor harbored CNVs gain. At least one sample had a gain that was defined as a subclonal gain. If all sample showed a loss or loss of heterozygosity (LOH), the tumor was considered as a clonal loss. Otherwise, the tumor was determined as a subclonal loss. The tumor purity for each sample was estimated by ABSOLUTE (v1.2)^34^.

### Tumor neoantigen detection

Tumor neoantigen was identified via netMHCpan (v4.0)^35^. Missense and nonsense mutations were correlated with the TNB counts using Spearman’s coefficient.

### Mutational signature analysis

The mutational signatures were analyzed using deconstructSigs (v1.8.0) and MutationalPatterns (v2.0.0)^36^. The mutational signature contribution for each patient was compared with COSMIC SBS signatureV2 (https://cancer.sanger.ac.uk/cosmic/signatures_v2.tt).

### Classification of driver genes, oncogene, and tumor suppressor genes

Genes in the COSMIC cancer gene census (https://cancer.sanger.ac.uk/cosmic) were defined as driver genes. The oncogene and tumor suppressor genes (TSG) were classified based on the driver gene list.

### Phylogenetic tree construction

All nonsilent somatic mutations excluding those co-localized within the LOH were used to construct phylogenetic trees via tools “ape” (v5.4-1), “phangorn” (v2.5.5), and “ggtree” (v2.2.4)^37^. Phylogenetic trees were built on the basis of the binary presence/absence matrices obtained from the regional distribution of variants within the tumor. Trunk mutations occurred in all regions of the tumor. The length of each tree’s branch was calculated according to the number of mutations on each branch.

### Cluster and timing of genomic alterations

All nonsilent somatic mutations were clustered by PyClone-VI (https://github.com/Roth-Lab/pyclone-vi)^38^ and corrected by copy number and purity. The number of clusters identified by PyClone was defined as mutation clusters. The average TMB in each mutation cluster identified by PyClone-VI was calculated as TMB/cluster.

The timing of SNVs was determined by EstimateClonality (v1.0)^10^. Briefly, we estimated the cellular prevalence of somatic mutations based on tumor purity and CNV and mutation copy number. Early mutations were defined as a mutation copy number of >1, whereas, late ones were classified as a mutation copy number of < = 1. The mutations in neutral copy numbers were clustered by sciClone (v1.1.0)^39^, then the results were used for evolution estimation through ClonEvol (v0.99.11)^40^ and plotted by fishplot^41^.

CNV gain was timed by the average mutation copy number of at least five mutations within each segment. The CNV gain was defined as “early” if the average mutation copy number was >1, and “late” if it was < = 1. Regarding CNV loss, clonal CNV loss coupled with genome doubling was classified as “early”, whereas, CNV loss unrelated to genome doubling was classified as “late”.

### ITH evaluation

Clonal SNV/indels were defined as mutations in the PyClone-VI cluster with a maximum cellular prevalence, while other SNV/indels in each tumor were defined as subclonal ones. SNV ITH was calculated by the number of subclonal mutations to all mutations.

CNV ITH was evaluated for each patient based on the presence of each CNV in different tumor regions with more than one variation and presented as the mean Jaccard distance among variation sets of each three regions^42^. ITH ranged from 0 to 1 (all branch events to all trunk events).

### Comparison with published multi-regional whole-exome sequencing data

To compare the genomic heterogeneity between SCLC and NSCLC, the multi-regional WES data for NSCLC of the TRACERx study was downloaded^10^, and the SNV ITH was recalculated for each sample using the same algorithm.

### Driver dominant score calculation

We calculated the driver dominant score, which measures the number of co-occurring drivers for each defined driver gene per tumor as Eq. (1)^33^. The ratio of patients carrying driver genes to the total number of patients was defined as an occurrence as Eq. (2). We downloaded the significant mutations for lung adenocarcinoma cancer (*n* = 10) and lung squamous cancer (*n* = 44)^43,44^. The driver genes were obtained from the mutation genes in lung adenocarcinoma and lung squamous cancers with *q* value < 0.1 by MutSig2CV results.

1 $${{{\rm{Dominant}}}}\,{{{\rm{score}}}}=\left(\right.{\sum }_{1}^{i}1/({{\rm{Frequency}}})\times 1/Frequency$$

2 $${{{\rm{Occurrence}}}}={{{\rm{Frequency}}}}/n$$

where *n* means the total number of patients of the cohort. The frequency represents the number of patients with the driver gene. *i* mean the number of driver genes.

### Statistical analysis

The Mann–Whitney–Wilcoxon test was used to compare the continuous numbers in different groups. Fisher’s exact test was performed to analyze differences between proportional data. The Kaplan–Meier curve between clinical features and survival was performed using “survminer” (v0.4.7) and “survival” (v3.2-10) packages. The cutoff values for the two groups were determined by the best cutoff point for each parameter, excluding TMB. TMB was classified by an upper quantile value in all patients (*n* = 40). The statistical significance was calculated using the Cox proportional hazards regression model and log-rank test for DFS and OS. All statistical analyses were performed with R v4.0.0 software. Statistical significance was defined as a two-sided *p* < 0.05.

### Reporting summary

Further information on research design is available in the Nature Research Reporting Summary linked to this article.

## Supplementary information

Supplementary Information

Description of Additional Supplementary Files

Supplementary Data 1

Supplementary Data 2

Supplementary Data 3

Supplementary Data 4

Supplementary Data 5

Supplementary Data 6

Reporting Summary

## Data Availability

The raw sequencing data generated in this study have been deposited in the GSA-Human (Genome Sequence Archive for Human in BIG Data Center, Beijing Institute of Genomics, Chinese Academy of Sciences, http://gsa.big.ac.cn/gsa-human) under the accession code HRA000441. The data are available under controlled access. Access to the data may be requested by completing the application form via GSA-Human System and is granted by the corresponding Data Access Committee. The approximate response time for accession requests is about 10 working days. Additional guidance can be found at the GSA-Human System website [https://ngdc.cncb.ac.cn/gsa-human/document/GSA-Human_Request_Guide_for_Users_us.pdf]. Public data used in this study include 1000 Genomes Project [https://www.internationalgenome.org/data-portal/data-collection/phase-3], HapMap3, dbSNP, and ExAC. TCGA mutation data were downloaded from https://www.cbioportal.org/datasets. TRACERx data can be obtained from https://www.cbioportal.org/study/summary?id=nsclc_tracerx_2017. The supplementary data of lung adenocarcinoma and lung squamous cancer can be obtained from https://www.nature.com/articles/nature13385 and https://www.nature.com/articles/nature11404, respectively. A complete list of somatic mutations and copy number variation can be found in Supplementary Data 2–5. Source data are provided with this paper. The data supporting Figs. 1, 2, 4, and 5 and Supplementary Figs. 1–4 of this study are available in the Source Data files. Source data are provided with this paper.

## Source data

Source Data

## Code availability

All custom code used in this work is available from https://github.com/LiyanJi-code/SCLC_MRS.
