# Supplementary material for: Multi-region exome sequencing reveals the intratumoral heterogeneity of surgically resected small cell lung cancer
Source: Nat Commun. 2021 Sep 14;12:5431. doi: 10.1038/s41467-021-25787-x (PMC8440529; doi:10.1038/s41467-021-25787-x)
Supplement: Supplementary file 1 — Supplementary Information [file 41467_2021_25787_MOESM1_ESM.pdf]

Multi-region exome sequencing reveals the intratumoral heterogeneity of surgically resected small cell lung cancer

Supplementary Information

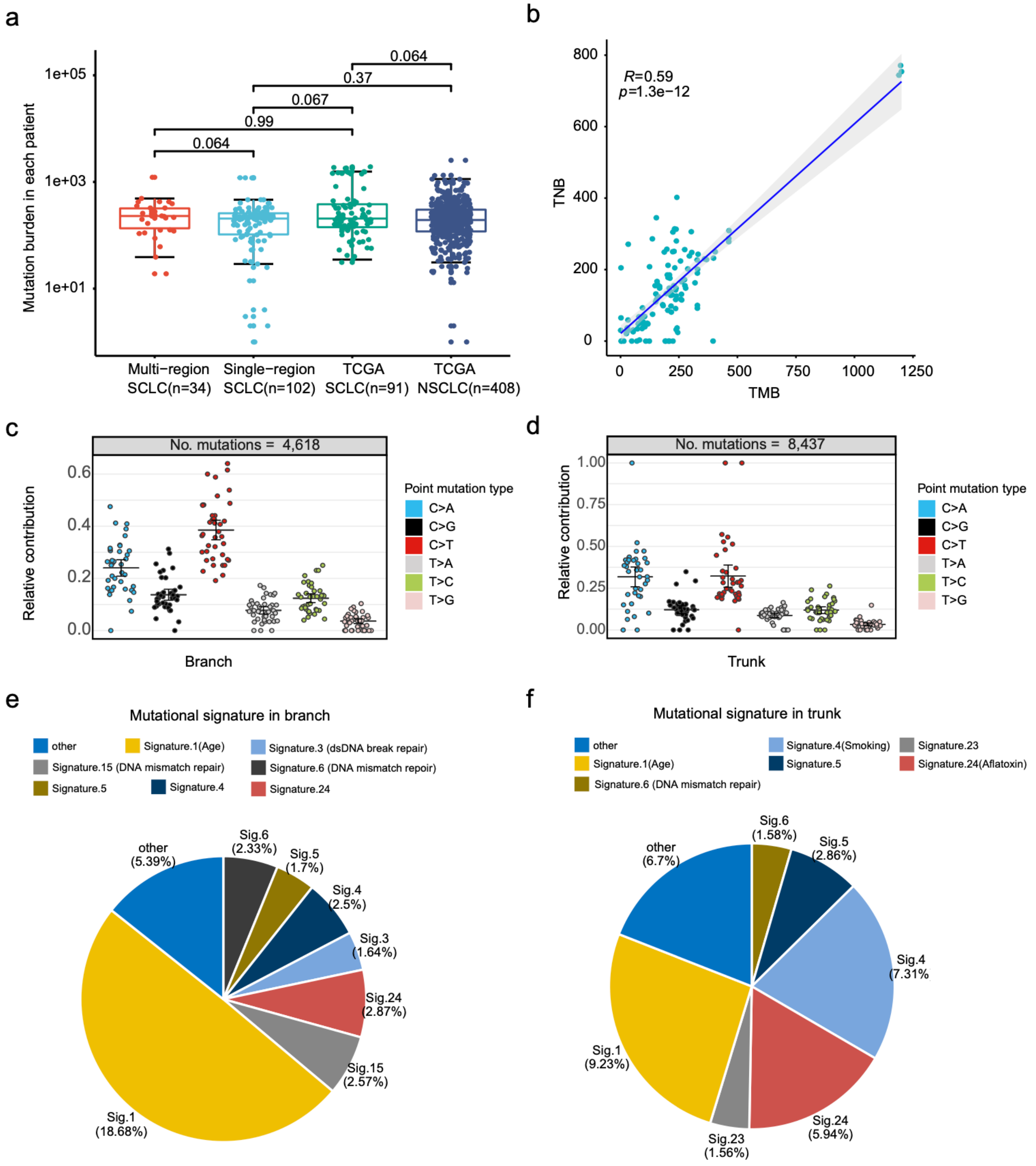

Supplementary Figure 1. **Tumor mutation burden and mutational signatures in SCLC**. (**a**) The TMB load in single- and multi-region exome sequencing in pure SCLC or NSCLC. *p* value from two-sided Mann-Whitney U-test. Boxplots are represented by center line, median; box limits, the 25th and 75th percentiles; whiskers extends represent the lower and upper values within 1.5 * inter-quartile range. (**b**) The correlations between TMB and tumor neoantigen burden using Spearman's coefficient. The estimated regression is shown as blue line with 95% confidence interval (gray shadow). The six base transversions and transitions in the branch (**c**) and trunk (**d**) mutations. Data are represented as mean values ± standard error of the mean. Pie plots depicting mutational signatures in the branch (**e**) and trunk (**f**). SCLC, small cell lung cancer; NSCLC, non-small cell lung cancer; TMB, tumor mutation burden; TNB, tumor neoantigen burden.

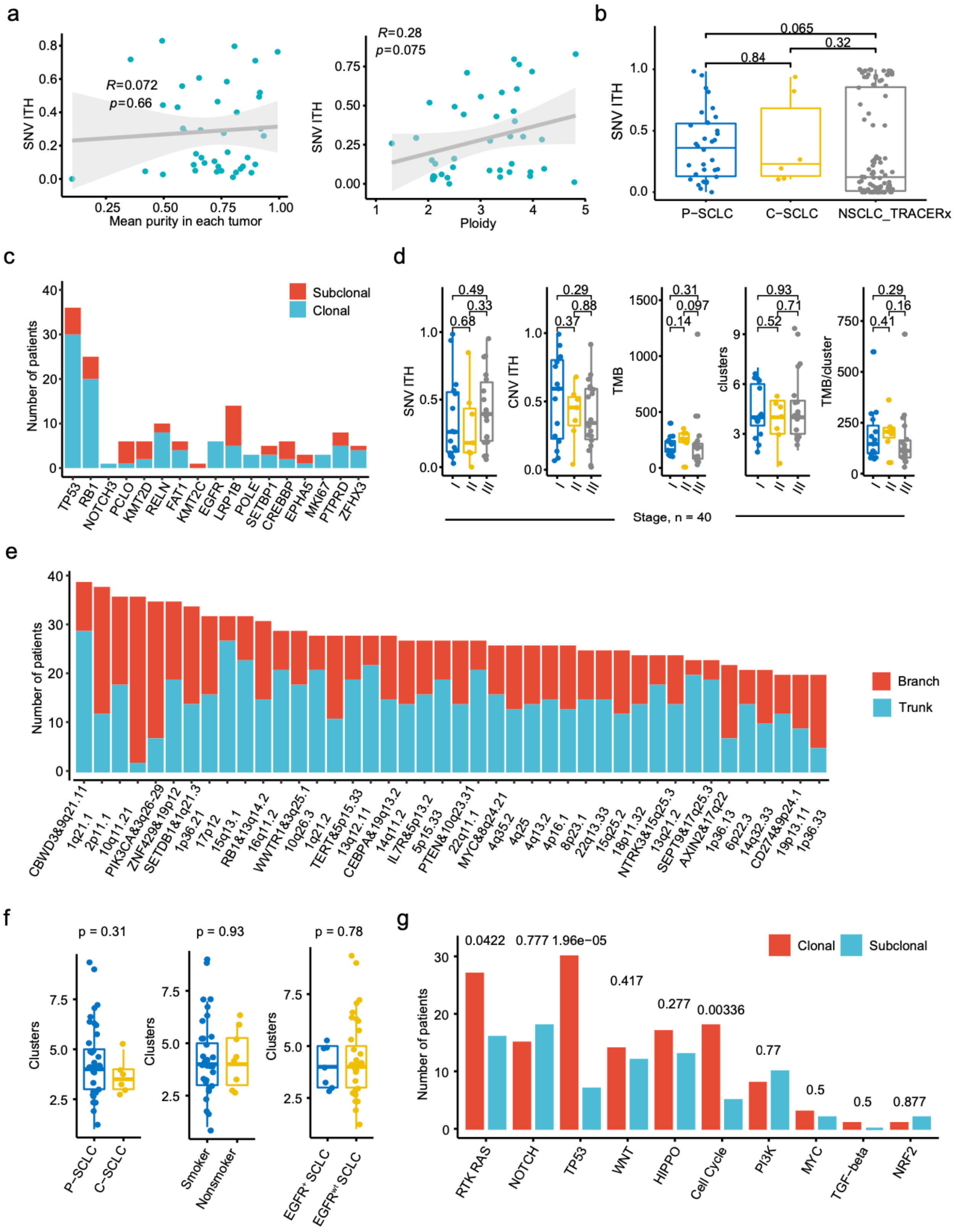
a
R=0.072
p=0.66
SNV ITH
Mean purity in each tumor
R=0.28
p=0.075
Ploidy
b
0.065
0.84
0.32
P−SCLC
C−SCLC
NSCLC_TRACERx
c
Subclonal
Clonal
Number of patients
TP53
RB1
NOTCH3
PCLO
KMT2D
RELN
FAT1
KMT2C
EGFR
LRP1B
POLE
SETBP1
CREBBP
EPHA5
MKI67
PTPRD
ZFHX3
d
SNV ITH
CNV ITH
TMB
clusters
TMB/cluster
Stage, n = 40
e
Branch
Trunk
CBWD3&9q21.11
1q21.1
2p11.1
10q11.21
PIK3CA&3q26-29
ZNF429&19p12
SETDB1&1q21.3
1p36.21
17p12
15q13.1
RB1&13q14.2
16q11.2
WWTR1&3q25.1
10q26.3
1q21.2
TERT&5p15.33
13q12.11
CEBPA&19q13.2
14q11.2
IL7R&5p13.2
5p15.33
PTEN&10q23.31
22q11.1
MYC&8q24.21
4q35.2
4q25
4q13.2
4p16.1
8p23.1
22q13.33
15q25.2
18p11.32
NTRK3&15q25.3
13q21.2
SEPT9&17q25.3
AXIN2&17q22
1p36.13
6p22.3
14q32.33
CD274&9p24.1
19p13.11
1p36.33
f
p = 0.31
p = 0.93
p = 0.78
Clusters
P−SCLC
C−SCLC
Smoker
Nonsmoker
EGFR+ SCLC
EGFRwt SCLC
g
Clonal
Subclonal
Number of patients
0.0422
0.777
1.96e−05
0.417
0.277
0.00336
0.77
0.5
0.5
0.877
RTK RAS
NOTCH
TP53
WNT
HIPPO
Cell Cycle
PI3K
MYC
TGF−beta
NRF2

Supplementary Figure 2. (**a**) The correlation between SNV ITH and mean purity (left) and ploidy (right) in each tumor. The estimated regression is shown as gray line with 95% confidence interval (gray shadow). (**b**) The comparison of mutational ITH in our SCLC with previous reported multi-region sequencing cohorts. *p* value from two-sided Mann-Whitney U-test. Boxplots are represented by center line, median; box limits, the 25th and 75th percentiles; whiskers extends represent the lower and upper values within 1.5 * inter-quartile range. (**c**) The number of clonal and subclonal mutated genes identified by PyClone for each patient. (**d**) The relationship between ITH and disease stage. *p* value from two-sided Mann-Whitney U-test. Boxplots are represented by center line, median; box limits, the 25th and 75th percentiles; whiskers extends represent the lower and upper values within 1.5 * inter-quartile range. (**e**) The number of clonal and subclonal genes affected by CNVs for each patient. (**f**) The mutation cluster distribution in different subgroups. *p* value from two-sided Mann-Whitney U-test. Boxplots are represented by center line, median; box limits, the 25th and 75th percentiles; whiskers extends represent the lower and upper values within 1.5 * inter-quartile range. (**g**) The percentage of pathway enrichment in clonal and subclonal genes affected by mutations. *p* value from two-sided Fisher's exact test. SNV, single-nucleotide variant; CNV, copy number variation; ITH, intratumoral heterogeneity; P-SCLC, pure small cell lung cancer; C-SCLC, combined small cell lung cancer; NSCLC, non-small cell lung cancer, TMB, tumor mutation burden.

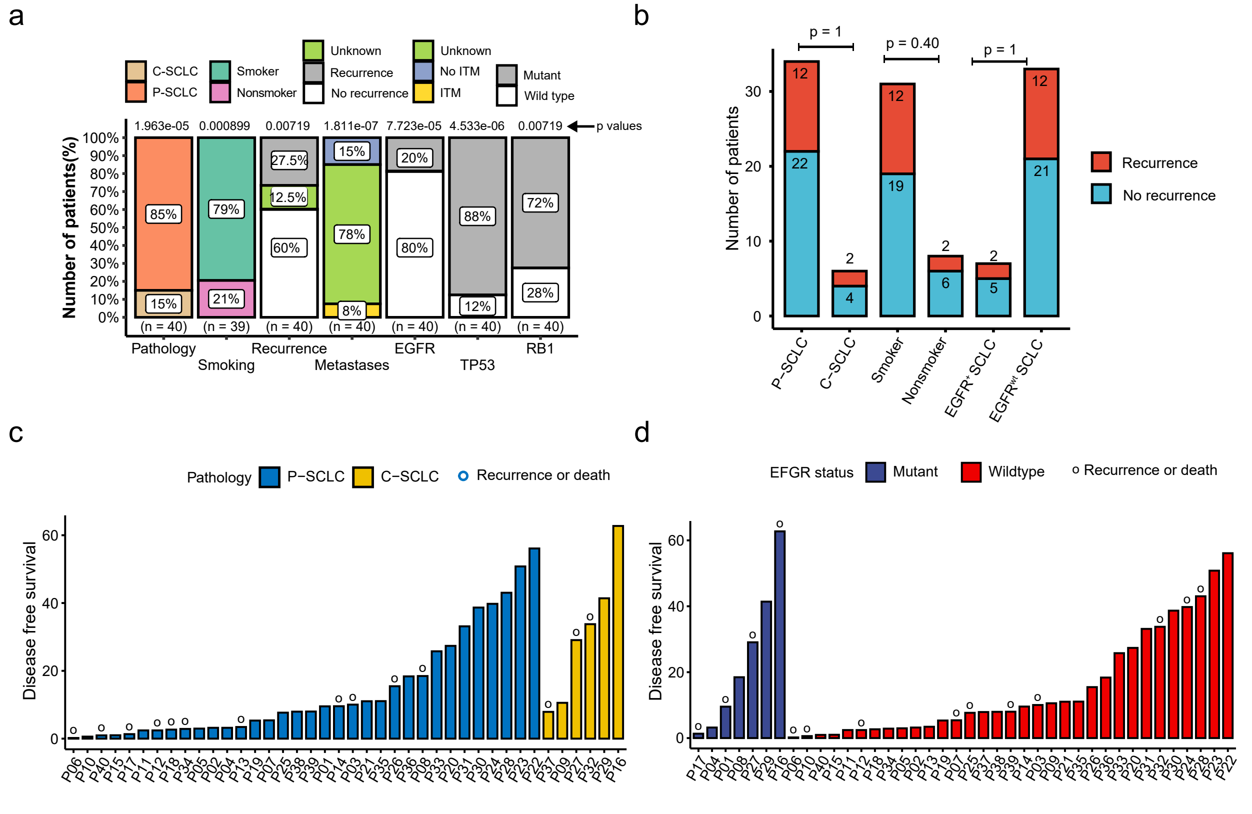

Supplementary Figure 3. **The basic clinical information of our SCLC cohort**. (**a**) The clinicopathological characteristics' distribution. For statistical comparison, *p* values were obtained from one sample proportion test. (**b**) The number of patients in subgroups stratified by recurrence or not. *p* values from two-sided Fisher's exact test. (**c**) Disease-free survival between P-SCLC and C-SCLC subgroups. The blue circles represent recurrence events. (**d**) Disease-free survival between EGFR mutant and wild type patients. The blue circles represent recurrence events. SCLC, small cell lung cancer; P-SCLC, pure small cell lung cancer; C-SCLC, combined small cell lung cancer; ITM, intrathoracic metastases; No-ITM, no intrathoracic metastases.

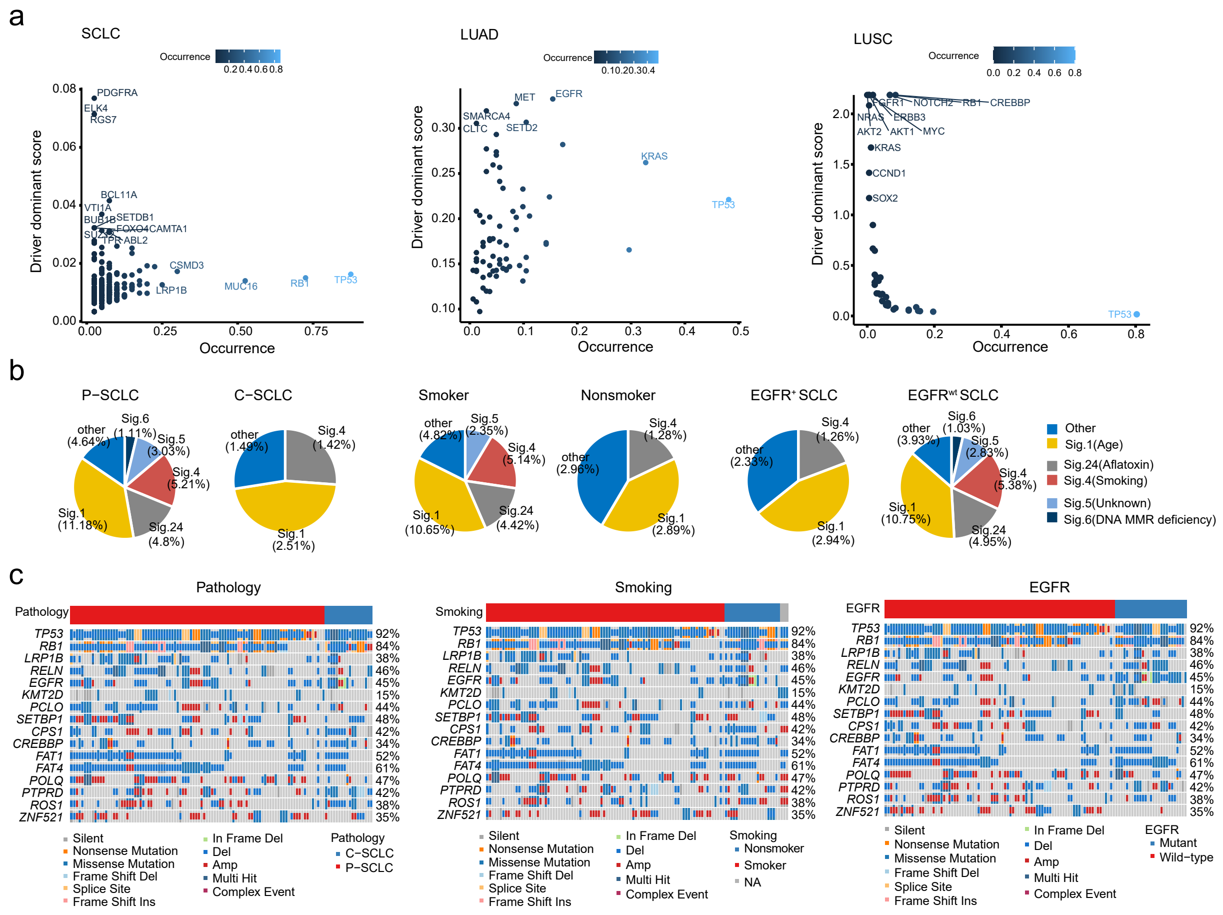

Supplementary Figure 4. **The gene mutation landscape of SCLC according to different subgroups**. (**a**) The driver dominant score in SCLC, LUAD and LUSC. (**b-c**) The gene signatures and mutations in different subgroups (pathology, smoking and EGFR). SCLC, small cell lung cancer; P-SCLC, pure small cell lung cancer; C-SCLC, combined small cell lung cancer; LUAD, lung adenocarcinoma; LUSC, lung squamous cell carcinoma.

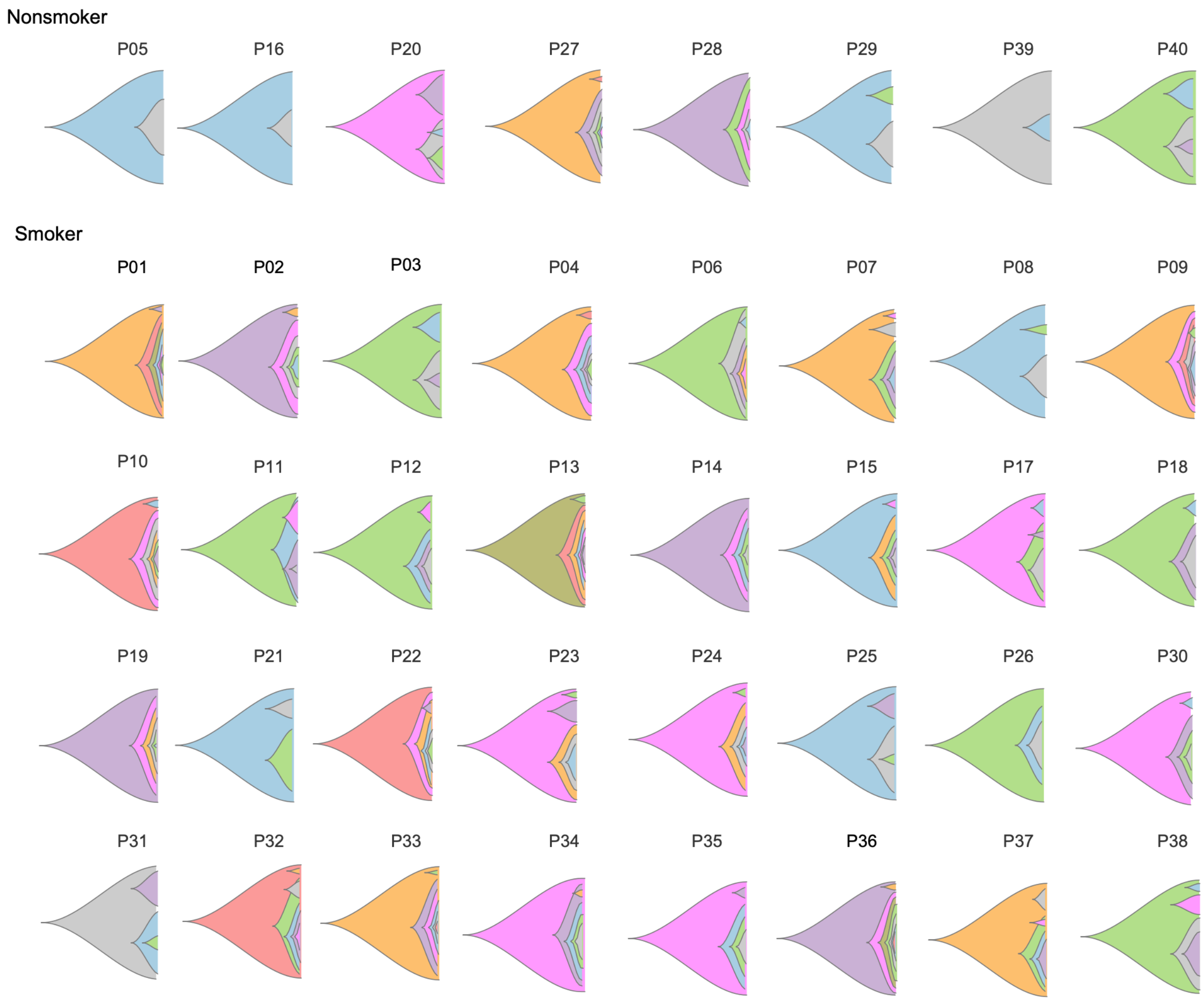

Supplementary Figure 5. **Phylogenetic trees and evolution in smoker and nonsmoker SCLC.** The fishplot depicting clonal structures inferred from somatic mutations at diploid by SciClone in each SCLC patients stratified by smoking. The different colors represent distinct clones.

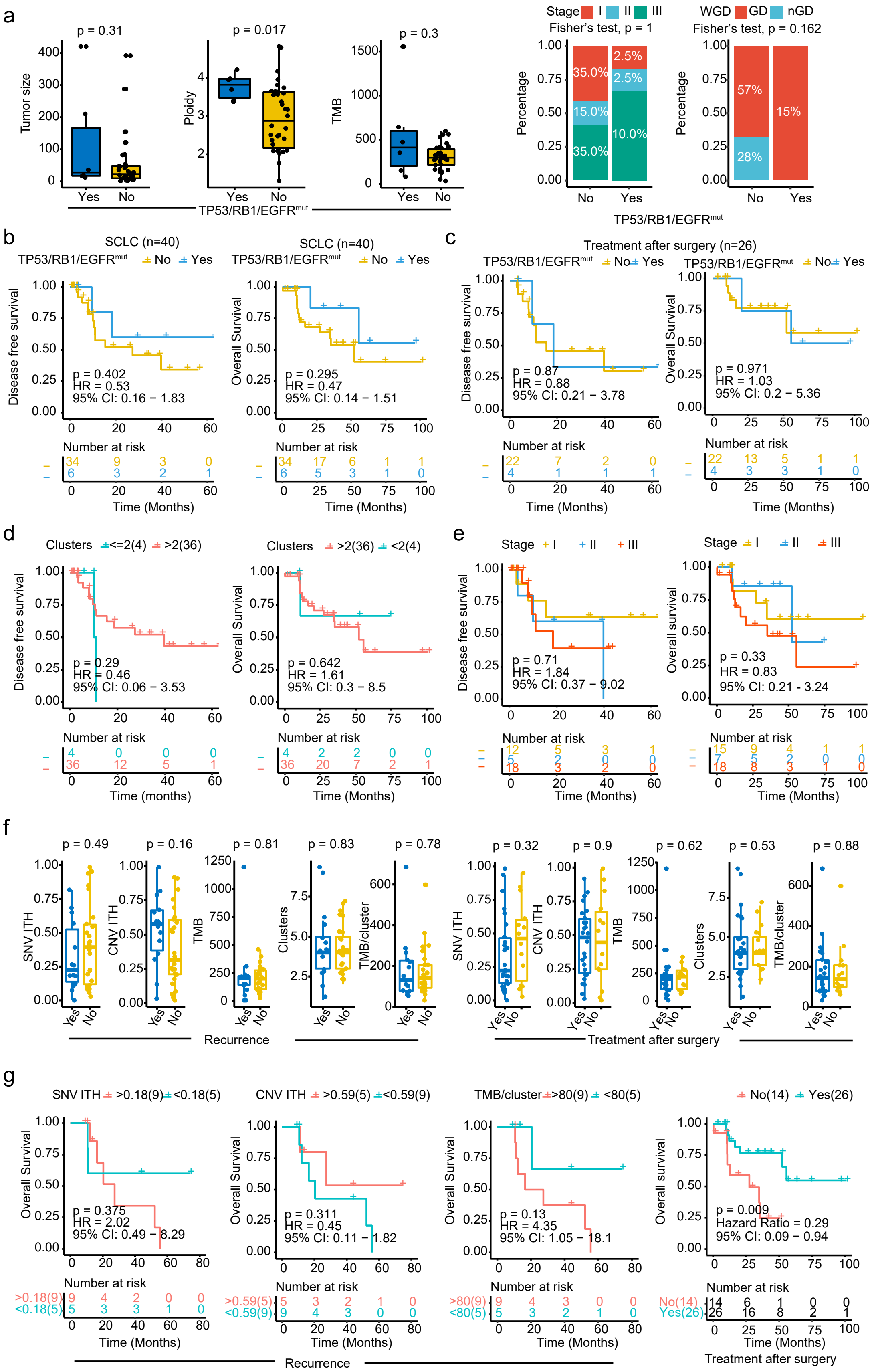
a
TP53/RB1/EGFRmut
b
SCLC (n=40)
c
Treatment after surgery (n=26)
d
e
f
Recurrence
Treatment after surgery
g
Recurrence
Treatment after surgery

Supplementary Figure 6. **Intratumor heterogeneity and outcomes in different subgroups of SCLC. (a)** Comparisons of tumor size, ploidy, TMB, stage and WGD in patients with or without TP53/RB1/EGFR mutations. *p* value from two-sided Mann-Whitney U-test. Boxplots are represented by center line, median; box limits, the 25th and 75th percentiles; whiskers extends represent the lower and upper values within 1.5 * inter-quartile range. **(b)** The Kaplan-Meier plot showing DFS and OS in patients with a TP53/RB1/EGFR mutation or not (n=40). **(c)** The DFS and OS survival analysis of patients who received systemic chemotherapy after surgery with a TP53/RB1/EGFR mutation or not (n=26). The outcome of patients stratified by **(d)** high or low mutation clusters and **(e)** stages. **(f)** The ITH in patients with or without recurrence (left) or treatment after surgery (right). *p* value from two-sided Mann-Whitney U-test. Boxplots are represented by center line, median; box limits, the 25th and 75th percentiles; whiskers extends represent the lower and upper values within 1.5 * inter-quartile range. **(g)** A Kaplan-Meier plot depicting overall survival in patients with recurrence or those treated after surgery and ITH. For all survival curves, the curves and statistical analysis are performed by Kaplan-Meier survival estimate and log rank test. WGD, whole-genome duplication; TMB, tumor mutation burden; SNV, single-nucleotide variant; CNV, copy number variation; ITH, intratumoral heterogeneity; SCLC, small cell lung cancer; P-SCLC, pure small cell lung cancer; C-SCLC, combined small cell lung cancer; HR, hazard ratio; CI, confidence interval. DFS, disease free survival; OS, overall survival.
