# Supplementary material for: Multi-region exome sequencing reveals the intratumoral heterogeneity of surgically resected small cell lung cancer
Source: Nat Commun. 2021 Sep 14;12:5431. doi: 10.1038/s41467-021-25787-x (PMC8440529; doi:10.1038/s41467-021-25787-x)
Supplement: Supplementary file 2 — Description of Additional Supplementary Files [file 41467_2021_25787_MOESM2_ESM.pdf]

Multi-region exome sequencing reveals the intratumoral heterogeneity of surgically resected small cell lung cancer

Description of Additional Supplementary Files

File Name: Supplementary Data 1

Description: Quality control of Whole Exome Sequencing in our SCLC cohort.

File Name: Supplementary Data 2

Description: Somatic mutation identified in the SCLC using multi-regional WES sequencing (n=40).

File Name: Supplementary Data 3

Description: Copy number variations in SCLC (n=40) identified using FACETS.

File Name: Supplementary Data 4

Description: Arm level Copy number variations in SCLC (n=40) identified by GISTIC2.

File Name: Supplementary Data 5

Description: Focal copy number variations in SCLC (n=40) identified by GISTIC2.

File Name: Supplementary Data 6

Description: TP53, RB1, and EGFR alterations in our cohort (n=40).
