# Supplementary material for: Multi-region exome sequencing reveals the intratumoral heterogeneity of surgically resected small cell lung cancer
Source: Nat Commun. 2021 Sep 14;12:5431. doi: 10.1038/s41467-021-25787-x (PMC8440529; doi:10.1038/s41467-021-25787-x)
Supplement: Supplementary file 9 — Reporting Summary [file 41467_2021_25787_MOESM9_ESM.pdf]

nature research

Corresponding author(s): Ningning Zhou

Last updated by author(s): Aug 8, 2021

Reporting Summary

Nature Research wishes to improve the reproducibility of the work that we publish. This form provides structure for consistency and transparency in reporting. For further information on Nature Research policies, see our Editorial Policies and the Editorial Policy Checklist.

Statistics

For all statistical analyses, confirm that the following items are present in the figure legend, table legend, main text, or Methods section.

n/a	Confirmed	
☐	☒	The exact sample size (*n*) for each experimental group/condition, given as a discrete number and unit of measurement
☐	☒	A statement on whether measurements were taken from distinct samples or whether the same sample was measured repeatedly
☐	☒	The statistical test(s) used AND whether they are one- or two-sided<br>*Only common tests should be described solely by name; describe more complex techniques in the Methods section.*
☐	☒	A description of all covariates tested
☐	☒	A description of any assumptions or corrections, such as tests of normality and adjustment for multiple comparisons
☐	☒	A full description of the statistical parameters including central tendency (e.g. means) or other basic estimates (e.g. regression coefficient) AND variation (e.g. standard deviation) or associated estimates of uncertainty (e.g. confidence intervals)
☐	☒	For null hypothesis testing, the test statistic (e.g. *F*, *t*, *r*) with confidence intervals, effect sizes, degrees of freedom and *P* value noted<br>*Give P values as exact values whenever suitable.*
☒	☐	For Bayesian analysis, information on the choice of priors and Markov chain Monte Carlo settings
☒	☐	For hierarchical and complex designs, identification of the appropriate level for tests and full reporting of outcomes
☒	☐	Estimates of effect sizes (e.g. Cohen's *d*, Pearson's *r*), indicating how they were calculated

Our web collection on statistics for biologists contains articles on many of the points above.

Software and code

Policy information about availability of computer code

Data collection	Data were collected in MS Office Excel (different versions)
Data analysis	The workflows of WES alignment, quality control and variant-calling algorithms are based on Sentieon-genomics pipeline (https://support.sentieon.com/appnotes/out_fields/#introduction). All custom code used in this work is available from https://github.com/LiyanJi-code/SCLC_MRS. The softwares used in this study include: Sentieon-genomics pipeline (version sentieon-genomics-201808); BWA MEM (v0.7.17-r1188); Sentieon-genomics TNscope (https://support.sentieon.com/appnotes/out_fields/#tnscope-reg) and MuTect2 software; Ensembl Variant Effect Predictor (VEP v93.3) ; MSIsensor tool (v0.2); FACETS (v0.5.11); ABSOLUTE (v1.2); GISTIC2.0; Integrative Genomics Viewer (v2.3.66); netMHCpan (v4.0); PyClone-VI (https://github.com/Roth-Lab/pyclone-vi); EstimateClonality (v1.0) ; R v4.0.0 software; “pheatmap” (v1.0.12); “maftools” (v2.6.05); “ape” (v5.4-1); “phangorn” (v2.5.5); “ggtree” (v2.2.4); “phytools” (v0.7-70); “tidytree” (v0.3.3); deconstructSigs (v1.8.0); MutationalPatterns (v2.0.0); sciClone (v1.1.0); ClonEvol (v0.99.11); “survminer” (v0.4.7); “survival” (v3.2-10); ”ggpubr”(v0.3.0).

For manuscripts utilizing custom algorithms or software that are central to the research but not yet described in published literature, software must be made available to editors and reviewers. We strongly encourage code deposition in a community repository (e.g. GitHub). See the Nature Research guidelines for submitting code & software for further information.

Data

Policy information about availability of data

All manuscripts must include a data availability statement. This statement should provide the following information, where applicable:

- Accession codes, unique identifiers, or web links for publicly available datasets
- A list of figures that have associated raw data
- A description of any restrictions on data availability

Public data used in this study include 1000 Genomes Project (https://www.internationalgenome.org/data-portal/data-collection/phase-3), HapMap 3 (https://www.sanger.ac.uk/resources/downloads/human/hapmap3.html), dbSNP (ftp://gsapubftp-anonymous@ftp.broadinstitute.org/bundle/b37/dbsnp_138.b37.vcf.gz), and ExAC (ftp://ftp.broadinstitute.org:/pub/ExAC_release/release0.3.1/subsets/ExAC_nonTCGA.r0.3.1.sites.vep.vcf.gz). TCGA mutation data were downloaded from https://www.cbioportal.org/datasets. TRACERx data can be obtained from https://www.cbioportal.org/study/summary?id=nsclc_tracerx_2017. The supplementary data of lung adenocarcinoma and lung squamous cancer can be obtained from https://www.nature.com/articles/nature13385 and https://www.nature.com/articles/nature11404, respectively. A complete list of somatic mutations and copy number variation can be found in Supplementary Data 2-5. The raw sequencing data generated in this study has been deposited in the GSA-Human (Genome Sequence Archive for Human in BIG Data Center, Beijing Institute of Genomics, Chinese Academy of Sciences, http://gsa.big.ac.cn/gsa-human) under accession code HRA000441 (https://ngdc.cncb.ac.cn/gsa-human/browse/HRA000441). Source data are provided with this paper. The data supporting Figures 1, 2, 4, and 5 and Supplementary Figures 1, 2, 3, and 4 of this study are available in the Source Data files.

Field-specific reporting

Please select the one below that is the best fit for your research. If you are not sure, read the appropriate sections before making your selection.

[x] Life sciences [ ] Behavioural & social sciences [ ] Ecological, evolutionary & environmental sciences

For a reference copy of the document with all sections, see nature.com/documents/nr-reporting-summary-flat.pdf

Life sciences study design

All studies must disclose on these points even when the disclosure is negative.

Sample size	No sample size calculation was performed. We collected 120 tumor samples from 40 stage I-III SCLC patients between September 2009 and September 2018. These sample sizes are sufficient for a descriptive study about SCLC-ITH.
Data exclusions	No data were excluded from the analyses.
Replication	In order to improve accuracy and reproducibility of the results we used two mutation calling algorithms for those somatic mutations to remove artifacts. This method has successfully identified the recurrent somatic gene alterations in SCLC samples.
Randomization	Treatment response, biomarkers were not the goal of this study and therefore randomization was not necessary.
Blinding	Treatment response, biomarkers were not the goal of this study and therefore blinding was not necessary.

Reporting for specific materials, systems and methods

We require information from authors about some types of materials, experimental systems and methods used in many studies. Here, indicate whether each material, system or method listed is relevant to your study. If you are not sure if a list item applies to your research, read the appropriate section before selecting a response.

Materials & experimental systems

n/a	Involved in the study	
[x]	[ ]	Antibodies
[x]	[ ]	Eukaryotic cell lines
[x]	[ ]	Palaeontology and archaeology
[x]	[ ]	Animals and other organisms
[ ]	[x]	Human research participants
[x]	[ ]	Clinical data
[x]	[ ]	Dual use research of concern

Methods

n/a	Involved in the study	
[x]	[ ]	ChIP-seq
[x]	[ ]	Flow cytometry
[x]	[ ]	MRI-based neuroimaging

Human research participants

Policy information about studies involving human research participants

Population characteristics	We included 40 surgically resected SCLC patients in this study, among them, 6 were diagnosed with combined SCLC (C-SCLC). Most SCLCs (34/40) were pure SCLC (P-SCLC). Table 1 shows the clinical characteristics of these patients. The median age was 62 years old. Most patients were male (35, 87.5%) and had a history of smoking (31, 77.5%). All patients underwent surgery, with a median tumor size of 22.5 mm. About 65% of patients received further treatment after surgery. Fifteen patients (15, 38%) died after a median follow-up time of 22.82 months.
Recruitment	Forty enrolled SCLC patients underwent thoracic surgery at Sun Yat-sen University Cancer Center between September 2009 and September 2018. There may be a selection bias, because patients who cannot provide enough sequenced samples cannot be included. But we have included all the specimens available in our center during this period, so it has little impact on the results.
Ethics oversight	The study protocol was approved by the institutional review board of Sun Yat-sen University Cancer Center. We have complied with all relevant ethical regulations for work with human participants, and that written informed consent was obtained.

Note that full information on the approval of the study protocol must also be provided in the manuscript.
